# Gastric Cancer Exosomes Trigger Differentiation of Umbilical Cord Derived Mesenchymal Stem Cells to Carcinoma-Associated Fibroblasts through TGF-β/Smad Pathway

**DOI:** 10.1371/journal.pone.0052465

**Published:** 2012-12-20

**Authors:** Jianmei Gu, Hui Qian, Li Shen, Xu Zhang, Wei Zhu, Ling Huang, Yongmin Yan, Fei Mao, Chonghui Zhao, Yunyan Shi, Wenrong Xu

**Affiliations:** 1 School of Medical Science and Laboratory Medicine, Jiangsu University, Zhenjiang, Jiangsu, China; 2 Department of Clinical Laboratory Medicine, The Affiliated Hospital of Jiangsu University, Zhenjiang, Jiangsu, China; University of Alabama at Birmingham, United States of America

## Abstract

**Background:**

Mesenchymal stem cells (MSCs) promote tumor growth by differentiating into carcinoma-associated fibroblasts (CAFs) and composing the tumor microenvironment. However, the mechanisms responsible for the transition of MSCs to CAFs are not well understood. Exosomes regulate cellular activities by mediating cell-cell communication. In this study, we aimed to investigate whether cancer cell-derived exosomes were involved in regulating the differentiation of human umbilical cord-derived MSCs (hucMSCs) to CAFs.

**Methodology/Principal Findings:**

We first showed that gastric cancer cell-derived exosomes induced the expression of CAF markers in hucMSCs. We then demonstrated that gastric cancer cell-derived exosomes stimulated the phosphorylation of Smad-2 in hucMSCs. We further confirmed that TGF-β receptor 1 kinase inhibitor attenuated Smad-2 phosphorylation and CAF marker expression in hucMSCs after exposure to gastric cancer cell-derived exosomes.

**Conclusion/Significance:**

Our results suggest that gastric cancer cells triggered the differentiation of hucMSCs to CAFs by exosomes-mediated TGF-β transfer and TGF-β/Smad pathway activation, which may represent a novel mechanism for MSCs to CAFs transition in cancer.

## Introduction

Tumor cells start to mold their microenvironment at early phase of the malignant progression [1]. Extensive reports have demonstrated the widespread interactions between tumor microenvironment and cancer cells, which are critical for tumorigenesis and tumor progression [2], [3]. Tumor microenvironment could elicit reversible changes in the phenotype of cancer cells and facilitate its metastatic spread [4], and changes of tumor microenvironment even dramatically affect the efficacy of cancer therapy. Tumor microenvironment is composed of diverse types of cells, including carcinoma-associated fibroblasts (CAFs), infiltrating immune cells, blood and lymphatic vascular networks, and mesenchymal stem cells (MSCs) [4], [5].

CAFs are key determinants in the malignant progression of cancer growth, vascularization, and metastasis. CAFs that express fibroblast activating protein (FAP) and α-smooth muscle actin (α-SMA) could create a niche for cancer cells and promote their motility [6], [7]. Indeed, CAFs undergo a differentiation process induced by tumor cells and develop invasive and migratory abilities [8], [9].

Mesenchymal stem cells are multipotent cells that can be isolated from a wide variety of tissues, including bone marrow, adipose tissue, synovium, skeletal muscle, liver, cord blood, placenta, and umbilical cord [10]–[13]. MSCs could be induced to differentiate into osteocytes, adipocytes, chondrocytes and myocytes because of their regenerative ability and multipotent capacity [14]. MSCs home to and survive at the sites of inflammation and injury as well as tumors, contributing to the formation of tumor-associated stroma [15]. Studies have confirmed that MSCs could differentiate into myofibroblasts, carcinoma-associated fibroblasts, fibrocytes or pericytes under the tumor microenvironment conditions [6], [16], [17]. In our previous studies, we have demonstrated that bone marrow MSCs and their derived exosomes could promote tumor growth [18], [19]. However, the mechanism responsible for this effect remains largely unknown.

Tumor cells interact with tumor microenvironment by cell-cell interaction and paracrine mechanisms such as producing a variety of growth factors, chemokines, and matrix-degrading enzymes that enhance the proliferation and invasion of the tumor [20]. In addition to the known mechanisms, a novel mechanism that tumor cells can actively release exosomes is emerging. Exosomes have a particular composition reﬂecting their origin and can transfer not only membrane components but also nucleic acid between different cells [21], [22], emphasizing their role in intercellular communication [23]. Accumulating evidence has shown the contribution of exosomes to a cellular mode of communication, leading to intercellular transfer of molecules [24]. Although the regulatory role of exosomes in the immune system and their application as vaccine in cancer immunotherapy is promising [25]–[28], the outcome following interaction between cancer exosomes and stromal cells is not well understood.

Malignant cells revert MSCs to CAFs that contribute to promote tumor progression has encouraged investigation into the possible mechanisms for its transition. Studies have demonstrated that at least 20% of CAFs originate from bone marrow and are derived from mesenchymal stem cells in mouse models of inﬂammation-induced gastric cancer [16]. We hypothesized that cancer cells communicated with MSCs through release of exosomes and transfer of proteins, therefore inducing the differentiation of MSCs to CAFs. In the current study, we demonstrated that MSCs acquired a CAF phenotype following exposure to cancer-derived exosomes and the differentiation of MSCs to CAFs was associated with the activation of TGF-β/Smad signaling pathway.

## Materials and Methods

### Cell Culture

Human umbilical cord MSCs were obtained as previously described [12], [29]. Fresh umbilical cords were collected from informed, consenting mothers and processed within 6 h. The cords were rinsed twice by phosphate-buffered saline (PBS) in penicillin and streptomycin and were then removed cord vessels. The washed cords were cut into pieces of 1–3 mm^2^-sized and floated in DMEM containing 10% FBS (Invitrogen, USA), 1% penicillin and streptomycin. The pieces of cord were subsequently incubated at 37°C in humid air with 5% CO_2_ and the medium was changed every 3 days after the initial plating. When well developed colonies of fibroblast-like cells reached 80% confluence, cultures were trypsinized and passaged into new flasks for further expansion. The characteristics of isolated hucMSCs including morphologic appearance, surface antigens, differentiation potential and gene expression were investigated as previously described [12]. All experiment protocols were approved by the Ethics Committee of Jiangsu University. The hucMSCs from passage 2 were selected for the experiments. Human gastric cancer cells (SGC7901 and HGC27) were purchased from Cell Bank, Type Culture Collection Committee (Chinese Academy of Sciences). Gastric epithelial cells (GES-1) were purchased from Cwbiotech Company and maintained in DMEM supplemented with 10% FBS.

### Exosome Isolation and Purification

SGC7901, HGC27 and gastric epithelial cells (GES-1) derived exosomes were isolated and purified as previously described [30], [31]. Briefly, cells were cultured in DMEM supplemented with 10% exosome-depleted fetal bovine serum. Fetal bovine exosomes were removed by overnight ultracentrifugation at 100,000 g for 16 h using a type 90 Ti fixed-Angel rotor (Optima L-90K, Beckman Coulter). Supernatants from confluent cultures (3–5 days) were centrifuged at 2000 g for 20 min to remove cells and debris. And the clarified supernatant was concentrated by ultrafiltration through a 100-kDa MWCO hollow fiber membrane (Millipore) at 1000 g for 30 min. The concentrated supernatant was loaded into centrifuge tubes (Beckman) and underlayed with 30% sucrose/D_2_O density cushion (5 ml) forming a visible interphase and ultracentrifuged at 100,000 g and 4°C for 1 h in a SW-32Ti swinging-bucket rotor (Optima L-90K, Beckman Coulter). Then both the exosomes sucrose density cushions (fraction 3) collected from the ultracentrifuge tubes bottom and the nonbanded fractions (fraction 6 and 7) which contain nonmembrane protein complexes collected for use as the exosomes control (E-control) were pooled and washed three times through a 100-kDa miniature hollow fiber cartridge (Millipore) at 1000 g for 30 min as described above. The protein content was measured using the BCA assay kit (Pierce). The exosomes were sterilized through a 0.22 µm capsule filter (Millipore) and stored at −70°C until use [30].

### Electron Microscopy

A drop of exosomes (about 20 µl) obtained after differential ultracentrifugation was pipetted onto formvar carbon-coated grids and allowed to stand for 1 min at room temperature. After removing the excess fluid with a piece of filter, the sample was stained with 2% (w/v) phosphotungstic acid (pH 6.8) for 5 min and was air-dried under an electric incandescent lamp, and analyzed with a transmission electron microscope (FEI Tecnai 12, Philips).

### Exosomes Labeling and Internalization

SGC7901 cells derived exosomes and the exosomes control were labeled with CM-Dil (red) according to the manufacturer’s protocol (Invitrogen). Exosomes from GES-1 cells were used as cell control. The labeled exosome suspension was filtered with a 100-kDa MWCO hollow fiber membrane (Millipore) and the flow-through was used as the unbound dye control. HucMSCs (1×10^4^/well) were seeded in 12-well plates containing lamellas and incubated at 37°C with labeled exosomes (800 µg/mL) for 4 h before harvest.

### Immunofluorescent Staining

HucMSCs were fixed in 4% paraformaldehyde for 10 min. Labeled cells were prepared for fluorescence microscopy by per-mobilization for 3 min with 0.1% Triton-X100, blocked with 5% BSA and incubated with rabbit monoclonal anti-β-actin antibody overnight, followed by incubation with Cy2-labeled anti-rabbit IgG secondary antibody at 37°C for 45 min (Jackson ImmunoResearch). The nuclei were counterstained with DAPI. Confocal images were sequentially acquired with TCS SP5 II system (Leica) [32].

### CAF Differentiation

HucMSCs were seeded in 6-well plates (5×10^3^/well). Twelve hours after seeding, hucMSCs were treated with exosomes (800 µg/mL) to trigger the CAF differentiation in the presence or absence of TGF-β receptor 1 kinase inhibitor SD208 (Sigma) or recombinant human TGF-β (5 ng/ml; Sigma). The medium was changed every 3 days for 14 days. Cells were then collected and prepared for Western blotting and quantitative PCR analysis.

### Western Blotting

Cells were collected and lysed with RIPA buffer (10 mM Tris, pH 7.4, 150 mM NaCl, 1 mM EGTA, 0.1% SDS, 1 mM NaF, 1 mM Na_3_VO_4_, 1 mM PMSF, 1 mg/mL aprotinin, 1 mg/mL leupeptin). Aliquots containing identical amounts of protein were fractionated and then transferred to methanol pre-activated PVDF membranes (Millipore, USA). After sequential incubation with the primary and secondary antibody, the signal was visualized using the HRP substrate (Millipore, USA) and analysed by MD Image Quant Software. Sources and dilution factors of primary antibodies were: rabbit polyclonal anti-CD9 (1∶1000; Bioworld), anti-CD81 (1∶1000; Epitomics), anti-TGF-β (1∶1000; Bioworld), anti-FAP (1∶1000, Abcam), mouse monoclonal anti-α-SMA (1∶1000; Santa Cruz), anti-VEGF (1∶1000; Santa Cruz) and anti-Vimentin (1∶2000; Santa Cruz), anti-N-cadherin (1∶1,000; Bioworld), anti-E-cadherin (1∶500; SAB), and mouse monoclonal anti-GAPDH (1∶5000; Kangcheng).

### Quantitative RT-PCR

Total RNA was extracted with the Trizol reagent (Invitrogen) and the cDNA was synthesized using a reverse transcription kit (Toyobo, Japan) according to the manufacturer’s instructions. Primers were produced by Invitrogen Company (Shanghai, China). Real-time RT-PCR was performed to detect the change of FAP, α-SMA, N-cadherin and IL-6 gene expression (Rotor-Gene 6000, Australia). To compensate for variations in input RNA and the efficiency of reverse transcription, an endogenous ‘housekeeping’ gene (β-actin) was also quantified to normalize the results. All samples were run in triplicate, and all reactions were repeated 3 times independently to ensure the reproducibility.

### Migration Assay

HucMSCs (8×10^4^ in 200 µL) suspended in serum-free medium were loaded into the upper compartment and 500 µL serum-free medium (SFM) containing 800 µg/mL exosomes in the presence or absence of TGF-β receptor 1 kinase inhibitor SD208 (2 µM) was added to the bottom of the transwell (Corning). After culture at 37°C for 6 hours, the cells upper the membrane were wiped with a cotton swab. The cells that had migrated through the membrane were fixed with 4% paraformaldehyde and stained with crystal violet. The cells were observed under microscopy and at least 10 fields of cells were assayed for each group.

### TGF-β Neutralization

HucMSCs were treated with tumor exosomes (800 µg/mL) to trigger the CAF differentiation in the presence or absence of TGF-β antibody (R&D). Prior to the experiments, anti-TGF-β antibody (20 µg/mL) was incubated with exosomes at 37°C for 2 hours.

### Statistical Analysis

All data were expressed as means ± SD. SPSS software was used to analyze the data. The means of different treatment groups were compared by two-way ANOVA test or Student’s t-test. P<0.05 was considered statistically significant.

## Results

### Exosomes Characterization and Internalization

**Figure 1:**
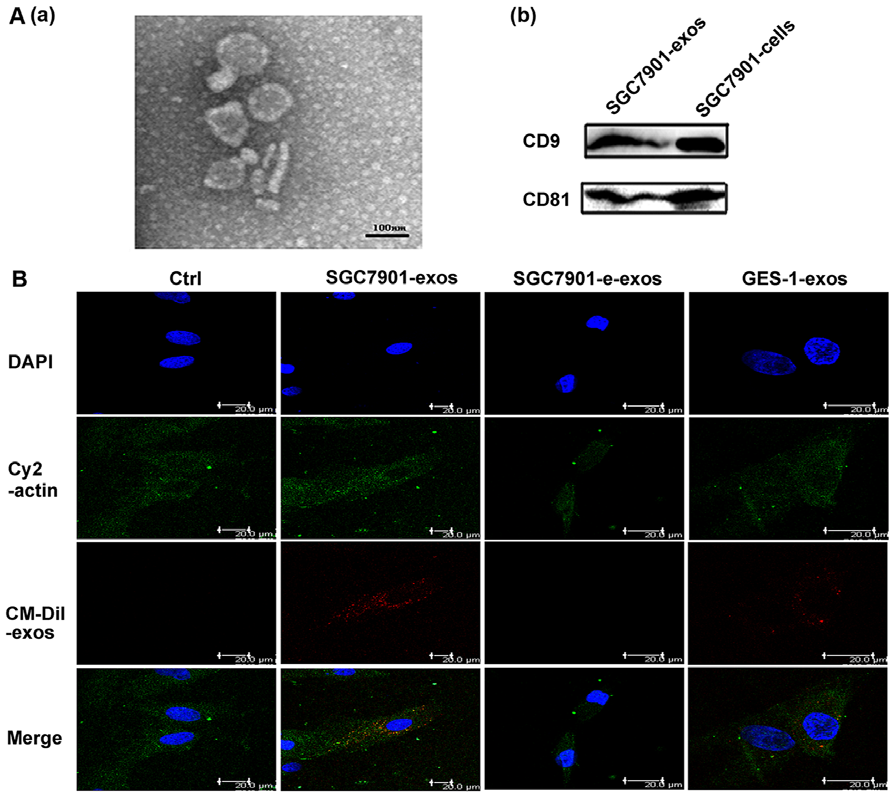
Exosomes characterization and MSC internalization. A. The morphology of exosomes and exosomal marker expression. (a) Morphologic analysis of gastric cancer derived exosomes. Scale bar = 100 nm. (b) CD9 and CD81 expression in exosomes. B. Exosomes were uptaken by hucMSCs. Exosomes labeled with CM-Dil (red) at 37°C for 1 h were added into hucMSCs and incubated for 4 h. Effluent from a filtered suspension of CM-Dil-labeled exosomes (control) and the CM-Dil-labeled withdrawn exosomes fraction (e-exos) were used as controls. Cells were fixed and stained for cytoplasm (cy2-actin, green) and nuclei (DAPI, blue). Scale bar = 20 µm.

We isolated and identified the exosomes based on their unique size and density. As shown in Fig. 1A–a, the exosomes had a characteristic saucer-like shape limited by a lipid bilayer with a diameter ranged from 40 to 100 nm. We confirmed the abundant expression of exosomal markers CD9 and CD81 in our isolated exosomes by Western blotting (Fig. 1A–b). Following our previous studies, human umbilical cord derived MSCs were prepared and characterized (data not shown). To investigate the internalization of exosomes by hucMSCs, we labeled the exosomes with CM-Dil. As shown in Figure 1B, SGC7901 cells derived exosomes were internalized and accumulated in hucMSCs after incubation for 4 hours while exosomes control showed minimal effect. Compared with SGC7901 group, less GES-1 derived exosomes were taken up by hucMSCs.

### Tumor-derived Exosomes Promote hucMSCs Differentiation to CAFs

**Figure 2:**
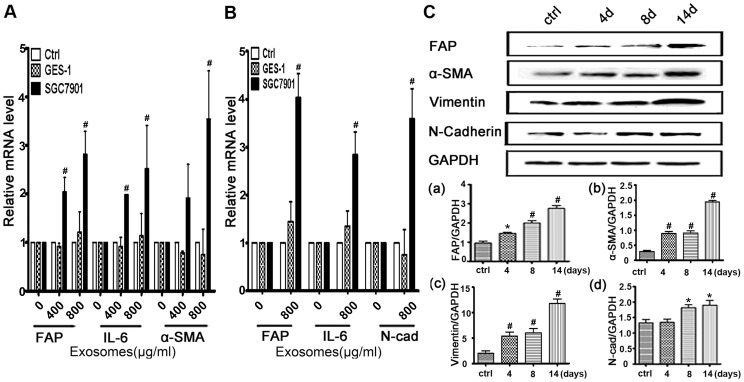
Gastric cancer cell derived exosomes induces the expression of CAF markers in hucMSCs. A.Quantitative analyses of the FAP, IL-6 and α-SMA mRNA expression in hucMSCs. HucMSCs were treated with various concentrations of SGC7901 exosomes or GES-1 exosomes for 36 hours. B. Quantitative analyses of the FAP, IL-6 and N-Cadherin expression in hucMSCs. HucMSCs were treated with various concentrations of SGC7901 exosomes or GES-1 exosomes for 14 days. C. Western blotting analyses of the FAP, α-SMA, N-Cadherin and Vimentin protein expression in hucMSCs treated with SGC7901 exosomes (800 µg/ml) for different times. (a–d) Density analysis of Western blotting bands. *P<0.05 and # P<0.01, compared to the relative control group (n = 3).

We hypothesized that tumor-derived exosomes might induce the differentiation of hucMSCs to CAFs *in vitro*. CAFs were defined as the increased expression of FAP and α-SMA proteins. HucMSCs monolayer was cultured in medium containing 800 µg/mL SGC7901 exosomes and GES-1 exosomes. Quantitative RT-PCR analyses showed that SGC7901 exosomes but not GES-1-exosomes promoted the expression of CAF markers in MSCs at 36 h (Fig. 2A) and 14 d (Fig. 2B) after induction, respectively. Compared to the untreated group, tumor exosomes treatment resulted in increases in FAP, α-SMA, N-cadherin, and Vimentin protein levels (Fig. 2C). Collectively, these results indicate that hucMSCs undergo CAF differentiation in response to tumor exosomes exposure.

### Tumor-derived Exosomes Promote hucMSCs Migration

**Figure 3:**
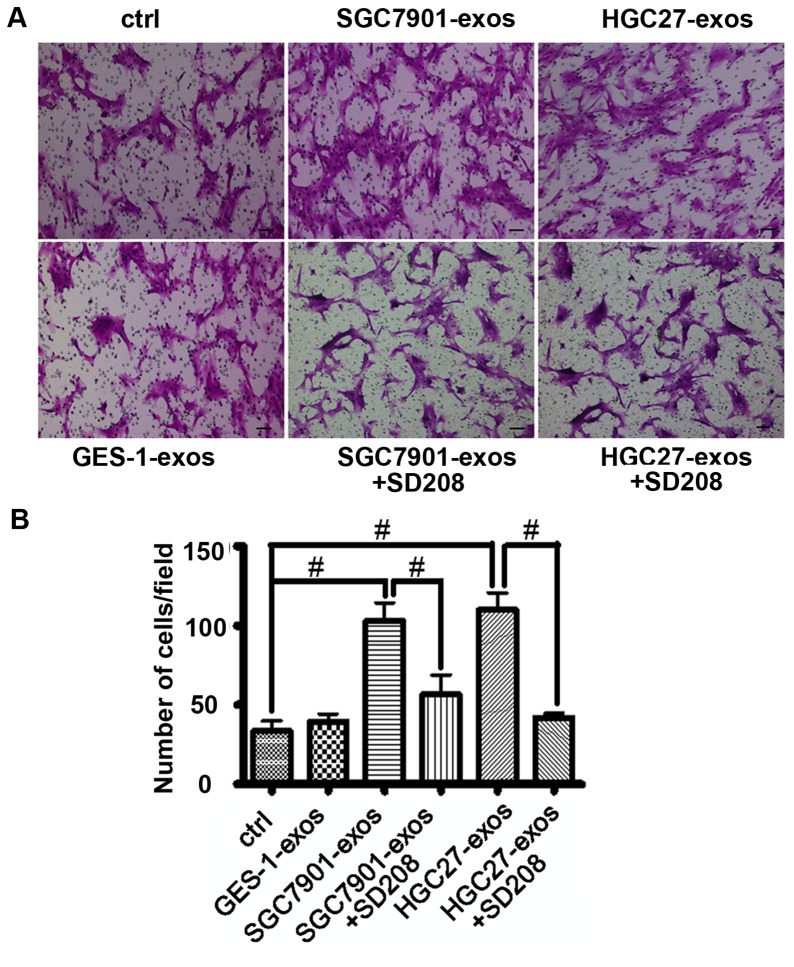
Gastric cancer cell derived exosomes promote hucMSCs migration. A. HucMSCs were treated with gastric cancer cell or normal gastric epithelial cell derived exosomes (800 µg/mL) in the presence or absence of SD208 (2 µM) for 6 h. Transwell migration assay was performed to analyze the migratory ability of the cells. B. The number of migrated cells above was evaluated. These experiments were repeated for three times. *P<0.05 and # P<0.01, n = 3. Scale bar = 50 µm.

To analyze whether the migratory ability of hucMSCs was affected by tumor exosomes, hucMSCs were cultured in the transwell and induced to migrate by tumor exosomes. As shown in Figures 3A and 3B, at 6 hours after incubation, tumor exosomes promoted the migration of hucMSCs more efficiently than normal cell derived exosomes. We also showed that the increased migration of hucMSCs by tumor exosomes was blocked by simultaneous treatment with TGF-β R1 inhibitor, SD208. Additionally, we examined the effect of SGC7901 exosomes on the migration of hucMSCs by using scratch assay. The results were consistent with that of transwell migration assay, showing a reduced gap distance in tumor exosome-treated group (Fig. S1). Taken together, these results indicate that tumor exosomes can promote the migration of hucMSCs *in vitro*.

### Tumor-derived Exosomes Activate Smad2/3 and p38 in hucMSCs

**Figure 4:**
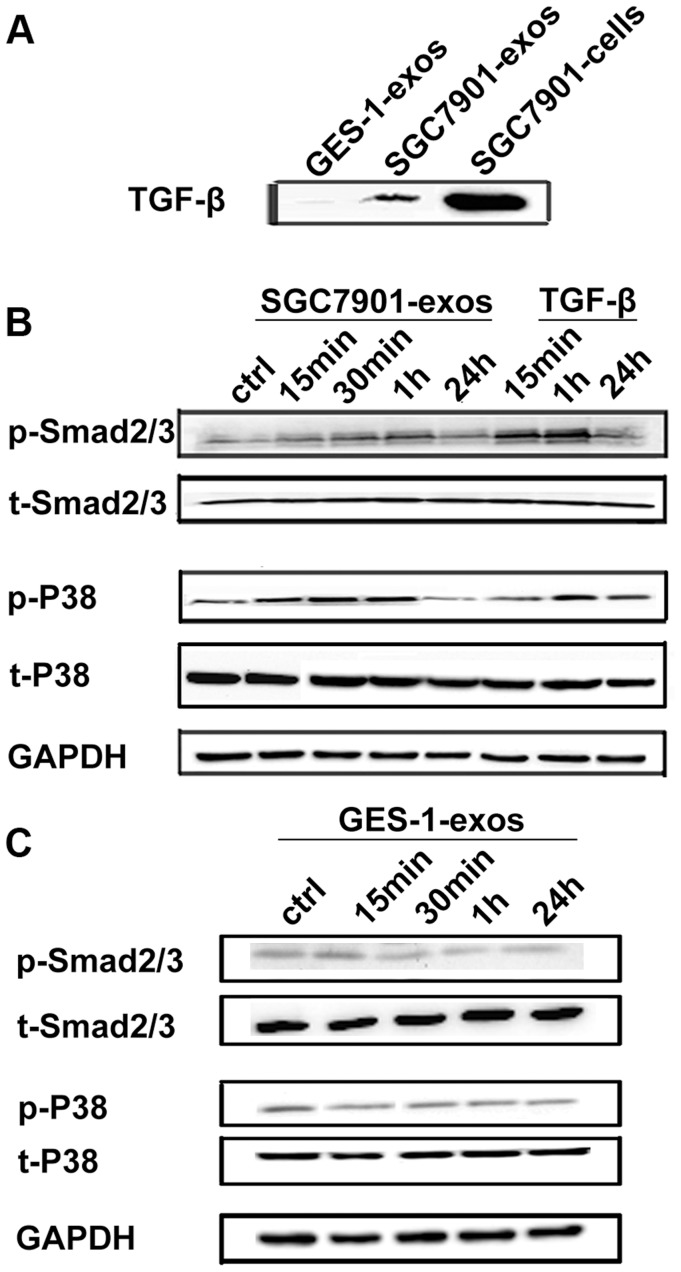
Gastric cancer cell derived exosomes induce Smad2/3 and p38 phosphorylation in hucMSCs. A. Western blotting analyses of TGF-β expression in gastric cancer cell (SGC7901) and normal gastric epithelial cell (GES-1) derived exosomes. B. HucMSCs were treated with SGC7901 derived exosomes (800 µg/mL) for different times as indicated. The levels of p-Smad2/3, t-Smad2/3, p-p38and t-p38 were analyzed by Western blotting. TGF-β served as a positive control. C. HucMSCs were treated with GES-1 derived exosomes (800 µg/mL) for different times as indicated. The levels of p-Smad2/3, t-Smad2/3, p-p38 and t-p38 were analyzed by Western blotting.

TGF-β has been shown to be present on the surface of exosomes [33] and critical for CAF differentiation. We then investigated whether TGF-β pathway was responsible for the induction of MSCs transition to CAFs by tumor exosomes. We first demonstrated the presence of TGF-β in tumor exosomes (Fig. 4A). Compared with tumor exosomes, normal cell derived exosomes showed lower level of TGF-β expression. To assess whether the differentiation of hucMSCs to CAFs is associated with TGF-β pathway activation, we examined the status of phosphorylated Smad 2/3 after tumor exosomes treatment. The results showed that Smad 2/3 phosphorylation was increased by tumor exosomes at 15 min post-treatment and reached the highest level at 60 min post-treatment, while the total Smad 2/3 protein levels were not affected. We also determined the level of phosphorylated p38 and found that p38 phosphorylation was also increased by tumor exosomes (Fig. 4B). In contrast, GES-1 derived exosomes could not activate Smad2/3 and p38 (Fig. 4C). Taken together, these results indicate that tumor exosomes specifically activate Smad2/3 and p38 in hucMSCs.

### TGF-β Pathway Inhibition Blocks Tumor Exosomes-induced Smad2/3 and p38 Activation

**Figure 5:**
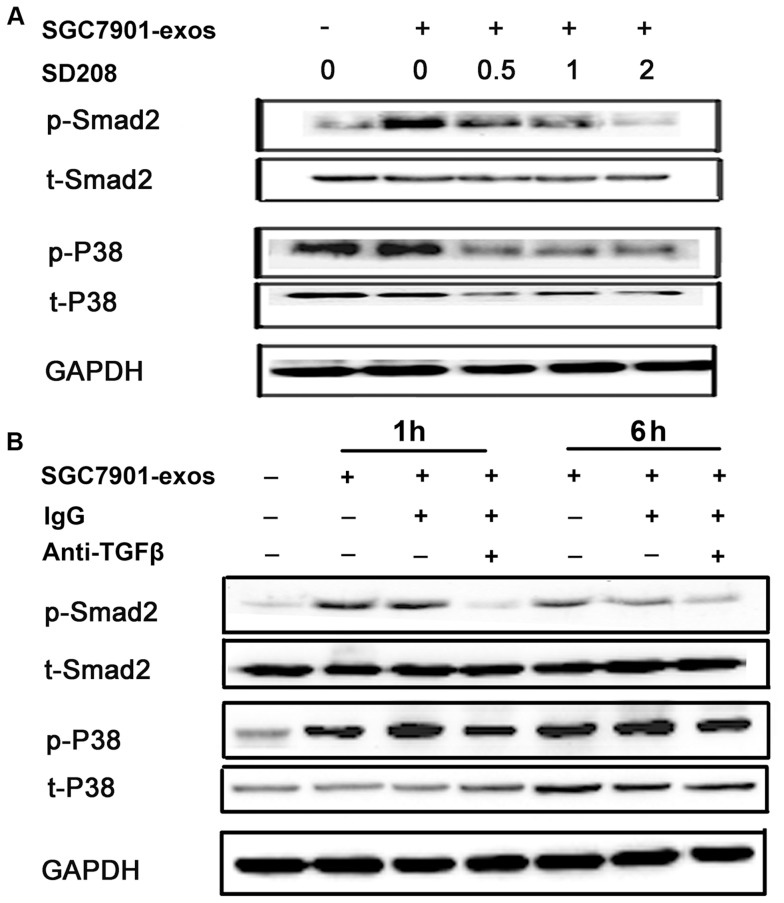
TGF-β inhibition reverses gastric cancer cell- exosomes induced Smad2 and p38 activation in hucMSCs. A. HucMSCs were treated with SGC7901 derived exosomes (800 µg/mL) in the presence or absence of SD208 (2 µM) for 1 hour. The levels of phosphorylated Smad2 and p38 were examined by Western blotting. B. Gastric cancer cell (SGC7901) derived exosomes were pre-incubated with anti-TGF-β antibody (20 µg/mL) for 2 h, and then added to hucMSCs. Six hours later, the cells were collected and the levels of phosphorylated Smad2 and p38 proteins were examined by Western blotting.

To demonstrate whether the activation of Smad2/3 and p38 is specific to TGF-β signaling triggered by tumor exosomes, we blocked TGF-β signaling pathway with TGF-β R1inhibitor and detected the levels of phosphorylated Smad2/3 and p38. As shown in Fig. 5A, the increased phosphorylation of Smad2/3 and p38 following tumor exosomes treatment was reversed by TGF-β R1 inhibitor, SD208, in a dose-dependent manner. To further demonstrate TGF-β from tumor exosomes that activates hucMSCs, we neutralized TGF-β in tumor exosomes using a anti-TGF-β antibody. Consistent with the results from TGF-β R1 inhibitor, the increased phosphorylation of Smad2/3 and p38 in hucMSCs were also reversed by TGF-β antibody (Fig. 5B). In summary, these data suggest that TGF-β from tumor exosomes interacts with TGF-β receptor on hucMSCs, leading to the sequential activation of Smad2/3 and p38 in hucMSCs.

### TGF-β Pathway Inhibition Reverses Tumor Exosomes-induced hucMSCs Differentiation to CAFs

**Figure 6:**
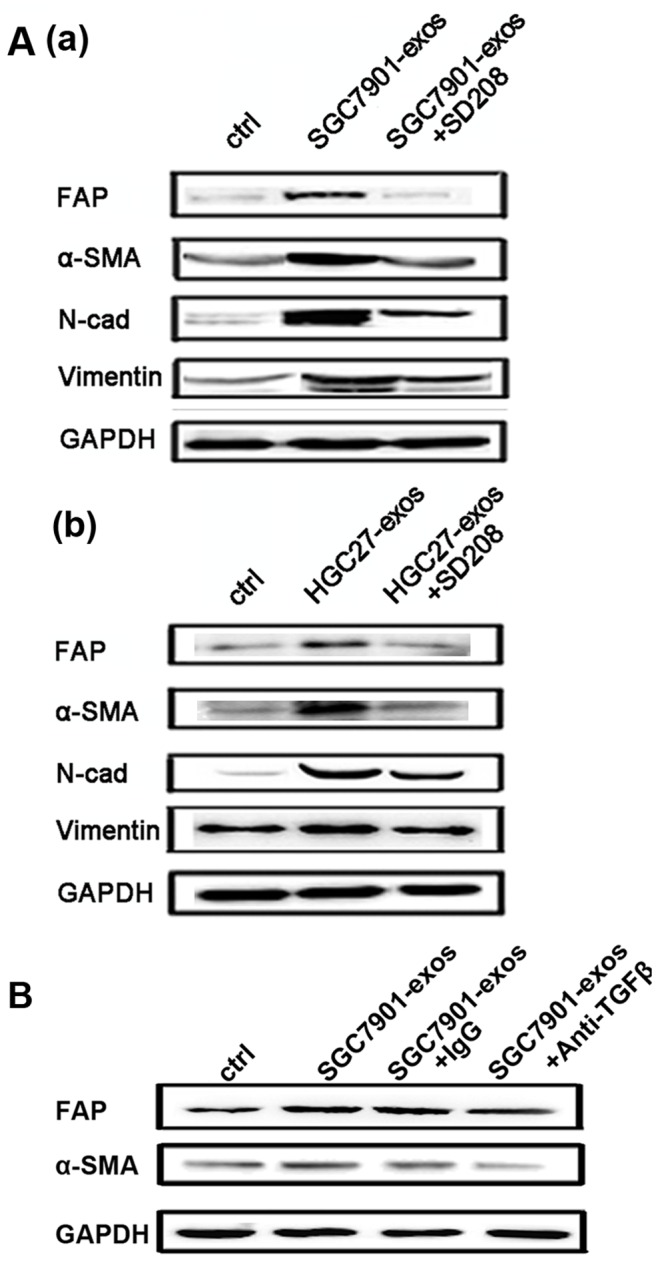
TGF-β inhibition attenuates gastric cancer cell derived exosomes-induced differentiation of hucMSCs to CAFs. A. HucMSCs were treated with gastric cancer cell derived exosomes in the presence or absence of SD208 (2 µM) for 2 weeks. The expression of FAP, α-SMA, Vimentin and N-cadherin proteins was determined by Western blotting. (a) SGC7901; (b) HGC27. B. HucMSCs were treated with gastric cancer cell (SGC7901) derived exosomes in the presence or absence of TGF-β antibody (20 µg/mL) for 2 weeks. Rabbit IgG was used as the control. The expression of FAP and α-SMA proteins was determined by Western blotting.

To demonstrate TGF-β/TGF-β R1 interaction is responsible for the differentiation of hucMSCs to CAFs induced by tumor exosomes, we blocked TGF-β signaling with SD208 and TGF-β neutralization antibody followed by tumor exosomes treatment. As shown in Figure 6A–a, treatment with SD208 (2 µM) for 14 days strongly reversed the tumor exosomes-induced expression of CAF markers including FAP, α-SMA, N-cadherin and Vimentin in hucMSCs. We also confirmed this effect by using exosomes from HGC-27 gastric cancer cells (Fig. 6A–b). In addition, treatment with anti-TGF-β antibody led to the similar effects to that observed in TGF-β R1 inhibitor (Fig. 6B). In summary, these data demonstrate that tumor exosomes mediate the differentiation of hucMSCs to CAFs through the activation of TGF-β/Smad signaling pathway.

## Discussion

In this study, we have identified a novel mechanism by which tumor cells induce the differentiation of MSCs to CAFs. Our data indicate that: (1) gastric cancer cell derived exosomes are internalized by hucMSCs; (2) gastric cancer cell derived exosomes trigger hucMSCs differentiation to CAFs and (3) TGF-β/Smad pathway mediates the transition of hucMSCs to CAFs. Our findings suggest that TGF-β in tumor exosomes may interact with the TGF-β R1 on hucMSCs, resulting in the activation of Smad pathway in hucMSCs and the subsequent differentiation of hucMSCs to CAFs.

Although it has been reported that tumor exosomes can improve the metastatic activity of tumor cells [34], the role of tumor exosomes in MSCs has not been revealed yet. Our findings demonstrate that tumor cells may regulate the mobility of MSCs, suggesting that tumor cells could recruit MSCs from bone marrow or other tissues to the tumor microenvironment by exosome-mediated mechanism.

Exposure to tumor exosomes increases the expression of CAF markers in MSCs, suggesting that tumor exosomes induces the switch of phenotype from MSCs to CAFs. While this paper was in preparation, Cho et al. reported that exosomes from breast and ovarian cancer cells could induce the adipose tissue derived MSCs to acquire physiological and functional characteristics of tumor-supporting myofibroblasts [35], [36]. Our work is in consistent with their findings and further demonstrates that this process is Smad-dependent.

In the current study, we present the evidence that tumor exosomes offer an efficient platform for transfer of specific messages to MSCs, promoting MSC-CAF transition. Several previous studies have shown that TGF-β is expressed by cancer cell-derived exosomes and this form of TGF-β is biologically active in driving Smad-dependent signaling [33], [37]. TGF-β is a key regulator of stem cell renewal and differentiation [38]. We verified the presence of TGF-β in gastric cancer cell derived exosomes and confirmed the TGF-β/TGF-β R1 interaction mediated the Smad2/3 activation and CAF differentiation in hucMSCs.

It has been demonstrated that MSCs could promote cancer metastasis [4], [39]. We have also reported that human MSC derived conditioned medium and exosomes enhance vascular endothelial growth factor (VEGF) expression in tumor cells and promote tumor growth *in vivo* [18], [19]. Whether the exosomes from MSCs could play a role in cancer metastasis has not been addressed. We have observed that MSCs exosomes promote the epithelial-to-mesenchyme transition (EMT) in gastric cancer cells, but the underlying mechanisms still needs to be investigated in the future studies.

In conclusion, we demonstrate in this study that gastric cancer cell derived exosomes have the capacity to induce the differentiation of MSCs to CAFs and the activation of TGF-β/Smad pathway by tumor exosomes contributes to the transition of MSCs to CAFs. Our findings provide a new mechanism by which tumor cells induce MSCs to differentiate into tumor stromal cells and participate in the formation of tumor microenvironment.

## Supporting Information

Figure S1 **Gastric cancer cell derived exosomes promote hucMSCs migration.** HucMSCs were treated with gastric cancer cell (SGC7901) derived exosomes (800 µg/mL). Scratch array was performed to analyze the migration ability of the cells. (a–c) Untreated hucMSCs; (d–f) SGC7901-exosomes treated hucMSCs. Scale bar = 50 µm.
(TIF)

## References

[1] Joyce JA (2005) Therapeutic targeting of the tumor microenvironment. Cancer Cell 7: 513–520. 15950901 10.1016/j.ccr.2005.05.024

[2] Bhowmick NA, Neilson EG, Moses HL (2004) Stromal fibroblasts in cancer initiation and progression. Nature 432: 332–337. 15549095 10.1038/nature03096 PMC3050735

[3] Hu M, Polyak K (2008) Microenvironmental regulation of cancer development. Curr Opin Genet Dev 18: 27–34. 18282701 10.1016/j.gde.2007.12.006 PMC2467152

[4] Karnoub AE, Dash AB, Vo AP, Sullivan A, Brooks MW, et al (2007) Mesenchymal stem cells within tumour stroma promote breast cancer metastasis. Nature 449: 557–563. 17914389 10.1038/nature06188

[5] Cao H, Xu W, Qian H, Zhu W, Yan Y, et al (2009) Mesenchymal stem cell-like cells derived from human gastric cancer tissues. Cancer Lett 274: 61–71. 18849111 10.1016/j.canlet.2008.08.036

[6] Spaeth EL, Dembinski JL, Sasser AK, Watson K, Klopp A, et al (2009) Mesenchymal stem cell transition to tumor-associated fibroblasts contributes to fibrovascular network expansion and tumor progression. PLoS One 4: e4992. 19352430 10.1371/journal.pone.0004992 PMC2661372

[7] Haubeiss S, Schmid JO, Murdter TE, Sonnenberg M, Friedel G, et al (2010) Dasatinib reverses cancer-associated fibroblasts (CAFs) from primary lung carcinomas to a phenotype comparable to that of normal fibroblasts. Mol Cancer 9: 168. 20579391 10.1186/1476-4598-9-168 PMC2907332

[8] Cat B, Stuhlmann D, Steinbrenner H, Alili L, Holtkotter O, et al (2006) Enhancement of tumor invasion depends on transdifferentiation of skin fibroblasts mediated by reactive oxygen species. J Cell Sci 119: 2727–2738. 16757516 10.1242/jcs.03011

[9] Giannoni E, Bianchini F, Masieri L, Serni S, Torre E, et al (2010) Reciprocal activation of prostate cancer cells and cancer-associated fibroblasts stimulates epithelial-mesenchymal transition and cancer stemness. Cancer Res 70: 6945–6956. 20699369 10.1158/0008-5472.CAN-10-0785

[10] Liechty KW, MacKenzie TC, Shaaban AF, Radu A, Moseley AM, et al (2000) Human mesenchymal stem cells engraft and demonstrate site-specific differentiation after in utero transplantation in sheep. Nat Med 6: 1282–1286. 11062543 10.1038/81395

[11] De Bruyn C, Najar M, Raicevic G, Meuleman N, Pieters K, et al (2011) A rapid, simple, and reproducible method for the isolation of mesenchymal stromal cells from Wharton's jelly without enzymatic treatment. Stem Cells Dev 20: 547–557. 20923277 10.1089/scd.2010.0260

[12] Qiao C, Xu W, Zhu W, Hu J, Qian H, et al (2008) Human mesenchymal stem cells isolated from the umbilical cord. Cell Biol Int 32: 8–15. 17904875 10.1016/j.cellbi.2007.08.002

[13] Gang EJ, Jeong JA, Hong SH, Hwang SH, Kim SW, et al (2004) Skeletal myogenic differentiation of mesenchymal stem cells isolated from human umbilical cord blood. Stem Cells 22: 617–624. 15277707 10.1634/stemcells.22-4-617

[14] Pittenger MF, Mackay AM, Beck SC, Jaiswal RK, Douglas R, et al (1999) Multilineage potential of adult human mesenchymal stem cells. Science 284: 143–147. 10102814 10.1126/science.284.5411.143

[15] Mishra PJ, Glod JW, Banerjee D (2009) Mesenchymal stem cells: flip side of the coin. Cancer Res 69: 1255–1258. 19208837 10.1158/0008-5472.CAN-08-3562

[16] Quante M, Tu SP, Tomita H, Gonda T, Wang SS, et al (2011) Bone marrow-derived myofibroblasts contribute to the mesenchymal stem cell niche and promote tumor growth. Cancer Cell 19: 257–272. 21316604 10.1016/j.ccr.2011.01.020 PMC3060401

[17] Ogawa M, LaRue AC, Drake CJ (2006) Hematopoietic origin of fibroblasts/myofibroblasts: Its pathophysiologic implications. Blood 108: 2893–2896. 16840726 10.1182/blood-2006-04-016600

[18] Zhu W, Huang L, Li Y, Zhang X, Gu J, et al (2012) Exosomes derived from human bone marrow mesenchymal stem cells promote tumor growth in vivo. Cancer Lett 315: 28–37. 22055459 10.1016/j.canlet.2011.10.002

[19] Zhu W, Huang L, Li Y, Qian H, Shan X, et al (2011) Mesenchymal stem cell-secreted soluble signaling molecules potentiate tumor growth. Cell Cycle 10: 3198–3207. 21900753 10.4161/cc.10.18.17638

[20] Egeblad M, Nakasone ES, Werb Z (2010) Tumors as organs: complex tissues that interface with the entire organism. Dev Cell 18: 884–901. 20627072 10.1016/j.devcel.2010.05.012 PMC2905377

[21] Gibbings DJ, Ciaudo C, Erhardt M, Voinnet O (2009) Multivesicular bodies associate with components of miRNA effector complexes and modulate miRNA activity. Nat Cell Biol 11: 1143–1149. 19684575 10.1038/ncb1929

[22] Valadi H, Ekstrom K, Bossios A, Sjostrand M, Lee JJ, et al (2007) Exosome-mediated transfer of mRNAs and microRNAs is a novel mechanism of genetic exchange between cells. Nat Cell Biol 9: 654–659. 17486113 10.1038/ncb1596

[23] Schorey JS, Bhatnagar S (2008) Exosome function: from tumor immunology to pathogen biology. Traffic 9: 871–881. 18331451 10.1111/j.1600-0854.2008.00734.x PMC3636814

[24] Fevrier B, Raposo G (2004) Exosomes: endosomal-derived vesicles shipping extracellular messages. Curr Opin Cell Biol 16: 415–421. 15261674 10.1016/j.ceb.2004.06.003

[25] Clayton A, Mitchell JP, Court J, Mason MD, Tabi Z (2007) Human tumor-derived exosomes selectively impair lymphocyte responses to interleukin-2. Cancer Res 67: 7458–7466. 17671216 10.1158/0008-5472.CAN-06-3456

[26] Liu C, Yu S, Zinn K, Wang J, Zhang L, et al (2006) Murine mammary carcinoma exosomes promote tumor growth by suppression of NK cell function. J Immunol 176: 1375–1385. 16424164 10.4049/jimmunol.176.3.1375

[27] Wolfers J, Lozier A, Raposo G, Regnault A, Thery C, et al (2001) Tumor-derived exosomes are a source of shared tumor rejection antigens for CTL cross-priming. Nat Med 7: 297–303. 11231627 10.1038/85438

[28] Valenti R, Huber V, Iero M, Filipazzi P, Parmiani G, et al (2007) Tumor-released microvesicles as vehicles of immunosuppression. Cancer Res 67: 2912–2915. 17409393 10.1158/0008-5472.CAN-07-0520

[29] Qian H, Yang H, Xu W, Yan Y, Chen Q, et al (2008) Bone marrow mesenchymal stem cells ameliorate rat acute renal failure by differentiation into renal tubular epithelial-like cells. Int J Mol Med 22: 325–332. 18698491

[30] Lamparski HG, Metha-Damani A, Yao JY, Patel S, Hsu DH, et al (2002) Production and characterization of clinical grade exosomes derived from dendritic cells. J Immunol Methods 270: 211–226. 12379326 10.1016/s0022-1759(02)00330-7

[31] Yu S, Liu C, Su K, Wang J, Liu Y, et al (2007) Tumor exosomes inhibit differentiation of bone marrow dendritic cells. J Immunol 178: 6867–6875. 17513735 10.4049/jimmunol.178.11.6867

[32] Mineo M, Garfield SH, Taverna S, Flugy A, De Leo G, et al (2012) Exosomes released by K562 chronic myeloid leukemia cells promote angiogenesis in a Src-dependent fashion. Angiogenesis 15: 33–45. 22203239 10.1007/s10456-011-9241-1 PMC3595015

[33] Webber J, Steadman R, Mason MD, Tabi Z, Clayton A (2010) Cancer exosomes trigger fibroblast to myofibroblast differentiation. Cancer Res 70: 9621–9630. 21098712 10.1158/0008-5472.CAN-10-1722

[34] Hao S, Ye Z, Li F, Meng Q, Qureshi M, et al (2006) Epigenetic transfer of metastatic activity by uptake of highly metastatic B16 melanoma cell-released exosomes. Exp Oncol 28: 126–131. 16837903

[35] Cho JA, Park H, Lim EH, Lee KW (2012) Exosomes from breast cancer cells can convert adipose tissue-derived mesenchymal stem cells into myofibroblast-like cells. Int J Oncol 40: 130–138. 21904773 10.3892/ijo.2011.1193

[36] Cho JA, Park H, Lim EH, Kim KH, Choi JS, et al (2011) Exosomes from ovarian cancer cells induce adipose tissue-derived mesenchymal stem cells to acquire the physical and functional characteristics of tumor-supporting myofibroblasts. Gynecol Oncol 123: 379–386. 21903249 10.1016/j.ygyno.2011.08.005

[37] Wang GJ, Liu Y, Qin A, Shah SV, Deng ZB, et al (2008) Thymus exosomes-like particles induce regulatory T cells. J Immunol 181: 5242–5248. 18832678 10.4049/jimmunol.181.8.5242 PMC4319673

[38] Mishra L, Derynck R, Mishra B (2005) Transforming growth factor-beta signaling in stem cells and cancer. Science 310: 68–71. 16210527 10.1126/science.1118389

[39] Albarenque SM, Zwacka RM, Mohr A (2011) Both human and mouse mesenchymal stem cells promote breast cancer metastasis. Stem Cell Res 7: 163–171. 21763624 10.1016/j.scr.2011.05.002

