# Supplementary figures and images for: Gastric Cancer Exosomes Trigger Differentiation of Umbilical Cord Derived Mesenchymal Stem Cells to Carcinoma-Associated Fibroblasts through TGF-β/Smad Pathway
Source: PLoS One. 2012 Dec 20;7(12):e52465. doi: 10.1371/journal.pone.0052465 (PMC3527492; doi:10.1371/journal.pone.0052465)

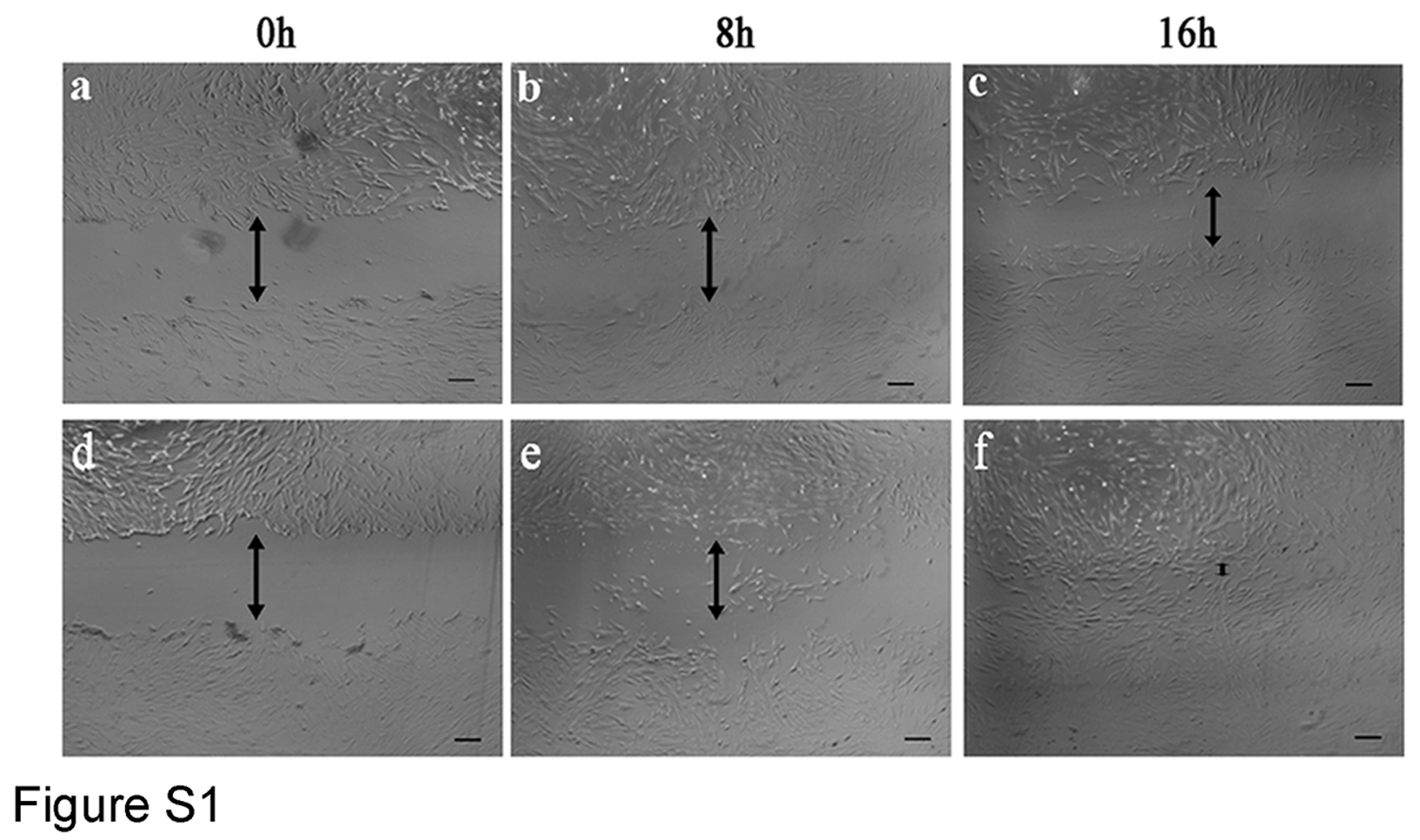

Supplement: Figure S1 — Gastric cancer cell derived exosomes promote hucMSCs migration. HucMSCs were treated with gastric cancer cell (SGC7901) derived exosomes (800 µg/mL). Scratch array was performed to analyze the migration ability of the cells. (a–c) Untreated hucMSCs; (d–f) SGC7901-exosomes treated hucMSCs. Scale bar = 50 µm. (TIF) [file pone.0052465.s001.tif]
